# Treatment and outcome of malignant bone tumors of the proximal humerus: biological versus endoprosthetic reconstruction

**DOI:** 10.1186/1471-2474-15-69

**Published:** 2014-03-07

**Authors:** Tang Liu, Qing Zhang, Xiaoning Guo, Xiangsheng Zhang, Zhihong Li, Xiaoyang Li

**Affiliations:** 1 Department of Orthopaedics, the Second Xiangya Hospital, Central South University, Changsha, Hunan 410011, P.R. China

**Keywords:** Malignant bone tumors, Humerus, Endoprosthetic reconstruction, Recycled pasteurized autograft

## Abstract

**Background:**

The purpose of this study was to compare the outcome, complications and survival of the commonly used surgical reconstructions of the proximal humerus after intrarticular tumour resection in our hospital.

**Methods:**

Between 1998 and 2010, 41 consecutive proximal humeral reconstructions using prosthesis (group P, n = 25) or recycled pasteurized autograft combined with non-vascularised fibula autograft (group B, n = 16) were performed.

**Results:**

The mean follow-up was 57.7 months. Fourteen patients (8 patients in group P and 6 in group B) died during the follow-up period, the disease-specific survival of patients in group P was 74.5% at 5 years and in group B was 67.0%. Local recurrences were occurred in 3 cases (12.0%) in group P and 2 (12.5%) in group B. Pulmonary metastases were observed in 4 patients (16.0%) in group P and 4 (25.0%) in group B. There was no significant difference in the incidence of local recurrence, pulmonary metastasis or death of disease. Revisions were indicated in 9 patients (36.0%) in group P and 5 (31.25%) in group B. Thought the incidence of revisions was higher in group P, there was no significant difference in these two groups. The Kaplan-Meier 5-year implant survival estimates, with revision for any reason as the end point, were 80.6% and 68.8% for group P and group B, respectively. The mean MSTS Score was 63.6% in group P and 63.0% in group B. These differences were not statistically significant.

**Conclusions:**

The study could show that prosthetic reconstruction and reconstruction with recycled pasteurized autograft are similar in terms of their local recurrence and metastasis, while the incidence of revisions was higher for patients with prosthetic reconstruction.

## Background

Malignant bone tumors of the proximal humerus are common, and multiple treatment options have been reported in recent years [1-11]. In most cases, surgery comprises an essential element of therapy. The clinical outcome, local recurrence, and survival rates after limb-preserving and other procedures seem to be comparable. To provide a platform for elbow and hand function, reconstructive limb-preserving procedures have been proposed for the proximal humerus. Moreover, patient acceptance has been described as higher for limb-preserving treatments. Therefore, amputation of the shoulder girdle is avoided if possible. In limb salvage procedures, large bone defects may result after resection of the proximal humerus. These defects can be reconstructed with autograft, allograft, implanted prostheses and composites [1-11].

Risks vary dependent on the choice of reconstruction. Biological reconstruction can be complicated by fracture, infection, and subchondral collapse, leading to implant revision or removal [1-11]. Difficulties with endoprosthetic reconstruction involve consequences of surgical resection of deltoid and rotator cuff. These include proximal subluxation, instability, and a reduction in functional range of motion [1-11]. The aim of this study was to investigate within a retrospective single center experience of surgically treated malignant bone tumors of the proximal humerus, respective oncological, surgical, and functional outcome differences after endoprosthetic and biological reconstruction in order to possibly identify predictive factors for appropriate indication.

## Methods

Between the year 1998 and 2010, 47 patients at our institution were treated for primary malignant bone tumor of the proximal humerus. Six of these 47 patients were lost to follow-up, so 41 patients were included in this study. There were 18 female and 23 male, with an average age of 30.6 years (range, 18 to 45 years) at the time of operation. The mean duration of follow-up was 57.7 months (range, 25 to125 months). Fourteen patients died during the follow-up period. This study was approved by the Second Xiangya Hospital committee for clinical research and informed consent was obtained from the patients participating in the study. The patients provided written informed consent for the publication of individual clinical details and accompanying images.

**Figure 1:**
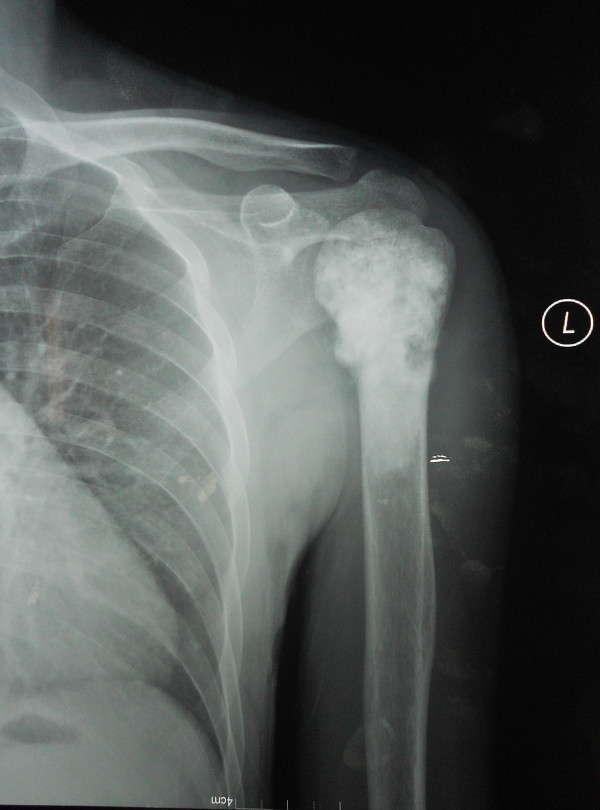
Preoperative X-ray (AP view) of a 39-year male patient with left humeral osteosarcoma.

**Figure 2:**
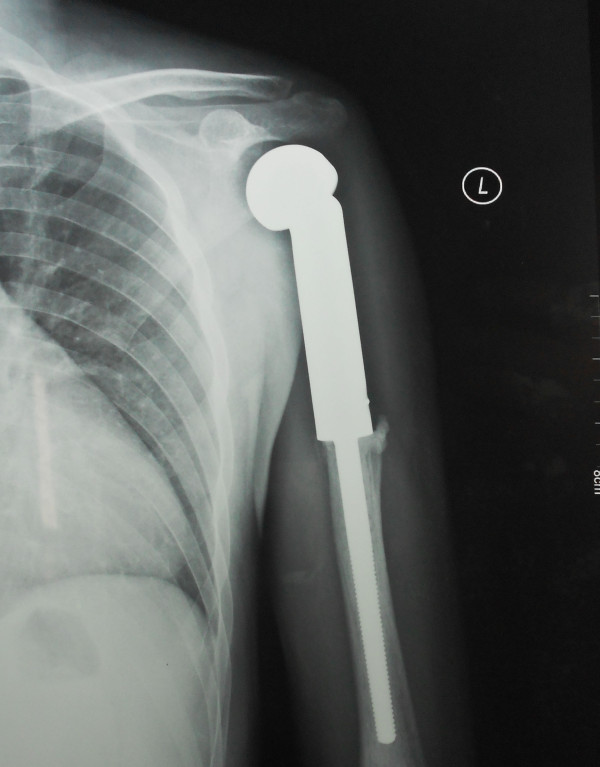
Post operative X-ray (AP view) showing 39 months follow-up of the same patient.

**Figure 3:**
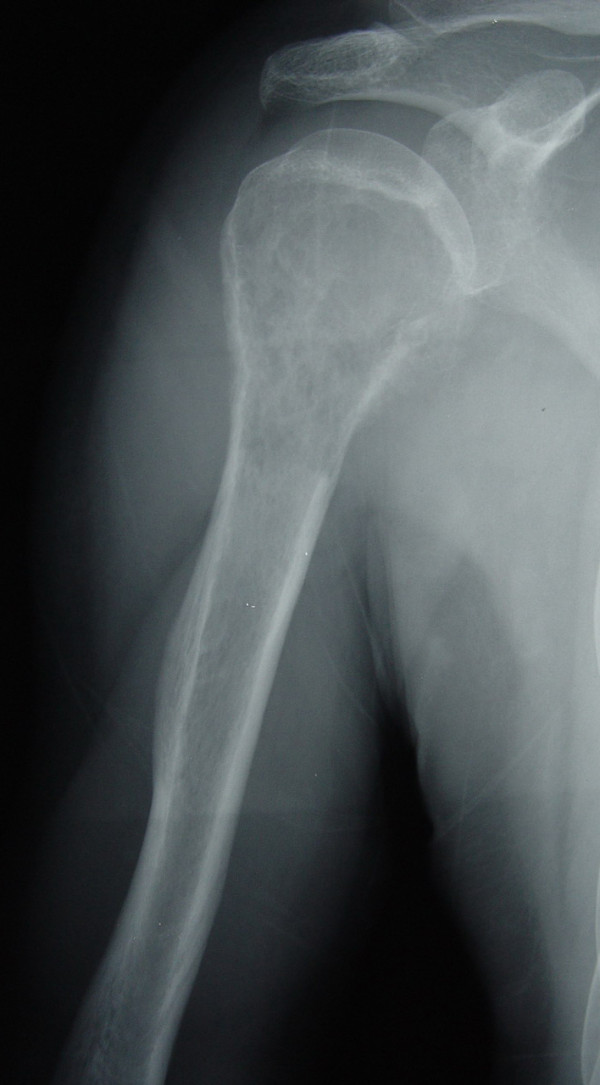
Preoperative X-ray (AP view) of a 35-year male patient with right humeral malignant fibrous histiocytoma.

**Figure 4:**
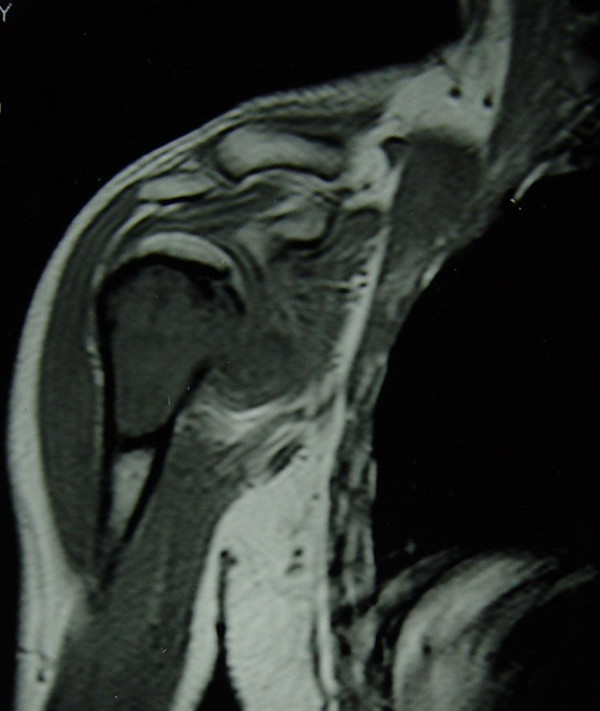
Preoperative MRI image of the same patient.

**Figure 5:**
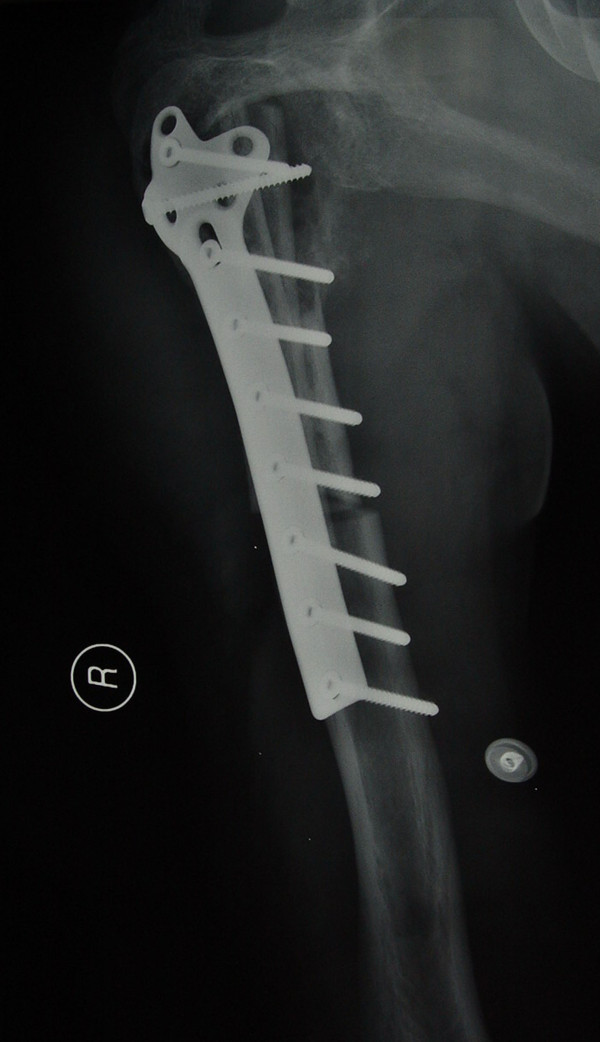
One week after reconstructions of the proximal humerus after intrarticular tumour resection by recycled pasteurized autograft combined with a non-vascularised fibula autograft.

**Figure 6:**
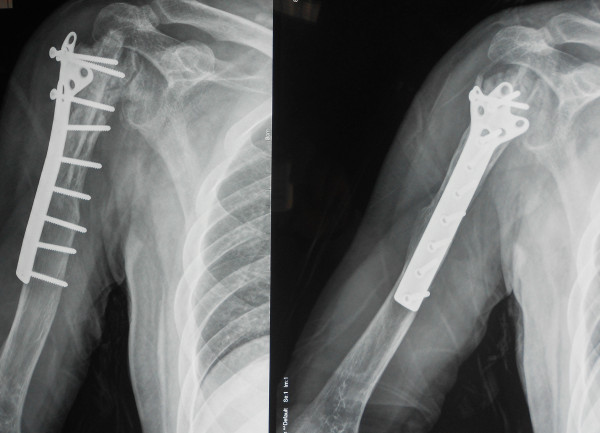
Post operative X-ray showing adequate union of the junction.

The histological diagnosis was osteosarcoma in 22 patients, Ewing’s sarcoma in 7, malignant fibrous histiocytoma in 9 and high-grade chondrosarcoma in 3. All these cases of bone tumors were staged according to Enneking’s criteria [12] with 19 stage IIA cases, and 22 stage IIB cases. All the patients underwent wide *en bloc* intra-articular excision and reconstruction of the defect with prosthesis in 25 cases (group P, see Figures 1 and 2) and biological means in 16 cases (group B). In the present study, biological reconstruction was used by recycled pasteurized autograft combined with a non-vascularised fibula autograft (see Figures 3, 4, 5 and 6). All patients with malignant tumors except chondrosarcoma received preoperative chemotherapies with a high dose of methotrexate, doxorubicin and cisplatin.

### Surgical technique

All patients were surgically treated by a single experienced musculoskeletal oncologist (Q Zhang). Except in rare instances in which biopsy sites or soft tissue extension of tumor was prohibitive, an anterior transdeltoid approach (paralleling the deltopectoral approach through the anterior 1/5 of the deltoid) was used and biopsy tracks, when present, were elliptically excised en bloc with the specimen. The humeral diaphysis was isolated and cut distally using an wire saw at a point at least 2.0 cm from the distal extent of the lesion. The lesion was measured on a coronal MR image from the tip of the greater tuberosity, which was localized intraoperatively through direct palpation or observation. If possible, based on the length of resection, the distal deltoid insertion was preserved. Cancellous bone was curetted from the medullary canal distal to the diaphyseal osteotomy and sent for intraoperative frozen section to confirm a negative margin. The humerus then was dissected circumferentially with a cuff of normal muscle tissue. Except in rare instances in which prior surgery or tumor proximity required sacrifice, the rotator cuff was preserved and released from near its insertions on the native humerus for later reattachment to the reconstruction. Likewise, the axillary nerve was preserved when practical. No other major nerves were sacrificed in any patient [1].

In group B, the mean length of the humeral defect after excision of the tumor segment was 10.88 cm (ranging from 8.0 to 13.5 cm). The bone was pasteurized in the following manner. After resection of the bone, soft tissue, gross tumor, and the intramedullary macroscopic portion of the tumor were cleared thoroughly from the specimen. It was then treated in saline, pre-heated at 65°C for 30-60 minutes. The pasteurized bone combined with a non-vascularised, autologous fibular graft were fixed by a plate and screws. In group P, the endoprosthesis used was a custom-made implant individually designed for each patient to replace the proximal of humerus (Li Dakang Co. Ltd, Beijng, China). The mean length of the humeral excision was 10.98 cm (ranging from 8.3 to 13.5 cm).

For evaluation of endoprosthetic and biological reconstruction methods, the two groups of patients were compared regarding their oncological, surgical, and functional outcome. For this, the post-operative events of infection, fracture, aseptic loosening, and nonunion have been noted as causes of further surgical revision. Functional outcome at latest clinical follow-up was evaluated applying the Musculoskeletal Tumor Society scoring system (MSTS Score) [13]. The patients were examined clinically and radiologically every month during the first six months after surgery to exclude the evidence of infection and local recurrence, every three months for the following two years and then every six months. A CT scan of the chest was performed every three months in the first year and then every six months to exclude pulmonary metastases. A bone scan was performed every six months in the first year and then annually until the last follow-up. Local recurrence was defined as re-occurrence of a tumor throughout this protocol at least four months after surgery.

### Statistical analysis

SPSS v13.0 (SPSS Inc., Chicago, Illinois) was used to perform the statistical analyses. Therapeutic variables (revision and function), pathological variables (Enneking stage, local recurrence and metastatic disease) and demographic variables (gender, age and duration of follow-up) were examined. The endpoints of the study were local recurrence, progression of disease and revision for any cause. Descriptive summary statistics included means and ranges. Age and time intervals were regarded as continuous variables. All other covariates were modeled as categorical variables. Differences between means and proportions were tested with the chi-squared test for categorical variables and the *t*-test for continuous variables. All tests were two-sided and a p-value < 0.05 was considered significant.

## Results

The detailed description of the results of all 41 patients were outlined in Table 1 and Table 2. The mean follow-up of group P is 55.08 months while the mean follow-up of group B is 61.75 months. In group B, all the patients had achieved bony union at the last follow-up and the mean time to graft union was 17.9 months (ranging from 12.5 to 27 month). Four patients (25.0%) acquired secondary iliac crest cancellous bone grafting to achieve union and one patient (6.25%) had a fracture because of slipping to the ground. This was changed the internal fixtor and the fracture subsequently united uneventfully. Local recurrences were occured in 3 cases (12.0%) in group P and 2 cases (12.5%) in group B. The 5 patients underwent subsequent amputation to treat their local recurrence. Pulmonary metastases were observed in 4 patients (16.0%) in group P and 4 patients (25.0%) in group B. Metastases were rated as irresectable due to dissemination and treated by further chemotherapy. These patients subsequently died of disease within 12 months. Table 3 summarizes the respective results and incidences of the two treatment groups with prosthetic and biological reconstruction. There was no significant difference in the incidence of local recurrence, pulmonary metastasis or death of disease (see Table 3). Superfical infection occurred in 1 patient (4.0%) in group P, which was resolved by debridements. There was no deep infection. Chondrolysis occcured in all the patients in group B and no treatment was required as painless in these patients.

**Table 1:** Patients’ data of biological reconstruction

**Case**	**Age(year)/gender**	**Ennecking’s stage**	**Specimen length (cm)**	**Local recurrence (months)**	**Pulmonary metastasis (months)**	**Revisions (number, months)**	**MSTS score (%)**	**Union time (months)**	**Follow-up time (months)**
1	21/F	Stage IIA	9.0	No	Conservative (19)	---	65	16.0	Death, 30
2	35/M	Stage IIB	10.0	No	No	---	71	17.5	Alive, 125
3	27/F	Stage IIB	9.5	No	No	Nonunion (1,13)	67	21.0	Alive, 109
4	23/F	Stage IIA	11.0	No	No	---	62	15.0	Alive, 97
5	33/M	Stage IIB	12.5	Amputation (21)	Conservative (27)	---	50	12.5	Death, 38
6	32/M	Stage IIB	8.3	No	No	Nonunion (1,10)	63	18.0	Alive, 71
7	39/F	Stage IIB	13.5	No	No	---	69	20.0	Alive, 85
8	31/M	Stage IIA	12.0	No	No	Fracture (1,17)	60	27.0	Alive, 72
9	45/F	Stage IIA	10.5	No	Conservative (15)	---	72	15.5	Death, 25
10	26/M	Stage IIB	12.0	No	No	Nonunion (1,12)	58	19.0	Death, 67
11	38/F	Stage IIB	9.5	No	No	---	67	13.5	Alive, 63
12	29/F	Stage IIB	11.5	No	No	---	65	22.0	Alive, 56
13	30/M	Stage IIA	12.0	No	No	---	55	18.0	Death, 50
14	42/M	Stage IIA	11.5	No	No	Nonunion (1,12)	67	19.5	Alive, 42
15	28/M	Stage IIB	9.7	Amputation (18)	No	---	52	16.0	Alive, 31
16	36/M	Stage IIB	11.5	No	Conservative (20)	---	65	15.5	Death, 27

**Table 2:** Patients’ data of prothesis

**Case**	**Age (year)/gender**	**Ennecking’s stage**	**Specimen length (cm)**	**Local recurrence (months)**	**Pulmonary metastasis (months)**	**Revisions (number, months)**	**MSTS score (%)**	**Follow-up time (months)**
1	29/M	Stage IIB	8.0	No	No	Aspetic loosening (1,68)	66	Alive, 120
2	37/M	Stage IIA	9.5	No	No	Aspetic loosening (1,89)	73	Alive, 112
3	26/F	Stage IIB	12.5	No	No	Aspetic loosening (1,76)	67	Alive, 105
4	18/M	Stage IIB	13.0	No	Conservative (20)	---	65	Death, 27
5	24/M	Stage IIB	13.5	No	No	---	65	Death, 76
6	41/F	Stage IIA	10.3	No	No	Aspetic loosening (1,73)	71	Alive, 82
7	36/M	Stage IIA	11.5	Amputation (16)	No	---	56	Death, 41
8	20/M	Stage IIB	12.5	No	No	Aspetic loosening (1,66)	67	Alive, 75
9	32/M	Stage IIB	12.8	No	Conservative (25)	---	60	Death, 34
10	28/F	Stage IIA	10.5	No	No	Aspetic loosening (1,61)	62	Death, 69
11	31/F	Stage IIB	8.5	No	No	---	67	Alive, 60
12	25/F	Stage IIB	10.5	No	No	Aspetic loosening (1,50)	61	Alive, 57
13	38/M	Stage IIB	10.0	Amputation (23)	No	---	51	Alive, 55
14	26/F	Stage IIA	9.5	No	No	---	58	Alive, 50
15	32/F	Stage IIA	11.0	No	No	---	63	Alive, 48
16	27/F	Stage IIB	9.5	No	Conservative (19)	---	72	Death, 26
17	31/M	Stage IIB	12.5	No	No	Aspetic loosening (1,43)	63	Alive, 47
18	38/M	Stage IIA	11.0	No	No	---	64	Alive, 45
19	29/F	Stage IIA	11.5	No	No	---	56	Alive, 41
20	31/F	Stage IIA	9.5	No	Conservative (32)	---	67	Death, 35
21	23/M	Stage IIB	10.2	No	No	Aspetic loosening (1,31)	62	Alive, 39
22	39/M	Stage IIB	11.3	No	No	---	67	Alive, 36
23	27/F	Stage IIB	10.0	Amputation (19)	No	---	53	Death, 32
24	25/M	Stage IIA	12.5	No	No	---	70	Alive, 35
25	28/F	Stage IIA	13.0	No	No	---	64	Alive, 30

**Table 3:** Main patient and outcome characteristics comparion

**Variable**	**Prothesis**	**Biological reconstruction**	***P-value***
Total	N = 25	N = 16	---
Mean age (years)	29.64 ± 5.99	32.19 ± 6.69	0.212^a^
Sex	13 M	9 M	0.790^b^
	12 F	7 F	
Mean follow-up (months)	55.08 ± 26.64	61.75 ± 30.42	0.464^a^
Ennecking’s Stage	Stage IIA 11	Stage IIA 6	0.680^b^
	Stage IIB 14	Stage IIB 10	
Specimen Length (cm)	10.98 ± 1.51	10.88 ± 1.43	0.820^a^
Local recurrence	3	2	0.962^b^
Metastasis	4	4	0.478^b^
Mean MSTS	63.6 ± 5.65	63.0 ± 6.48	0.756^a^
Revision	9	5	0.754^b^

^a^t-Test.

^b^Chi-square-test.

**Figure 7:**
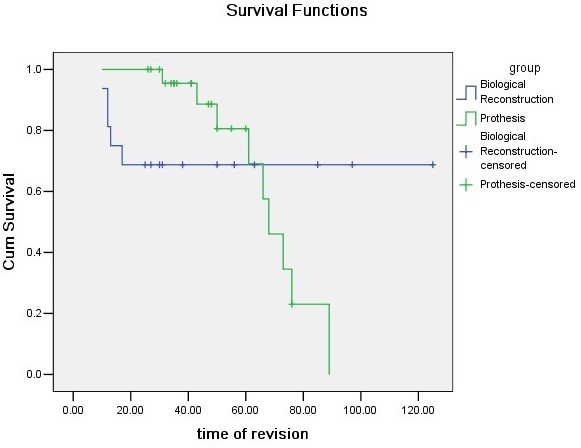
Kaplan-Meier implant survival curve subgroup analysis for the different types of reconstruction techniques with revision surgery as end point.

**Figure 8:**
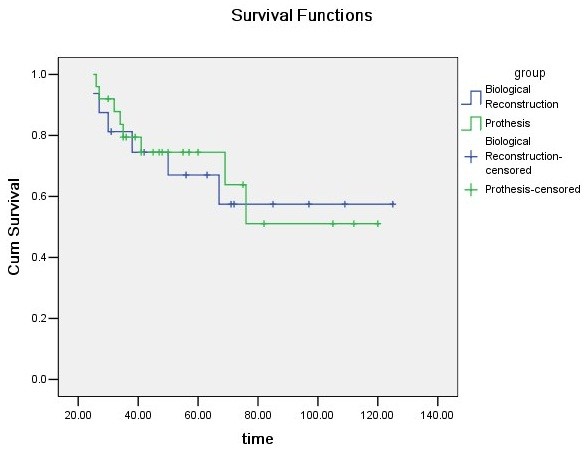
Kaplan-Meier disease-specific survival of patients.

Revisions were indicated in 9 patients (36.0%) in group P and 5 patients (31.25%) in group B. The incidence of revisions was higher for patients with prosthetic reconstruction. However, there was no significant difference regarding revision in these two groups (see Table 3). The Kaplan-Meier 5-year implant survival estimates, with revision for any reason as the end point, were 80.6% and 68.8% for the group P and group B, respectively (Figure 7). All patients were functionally assessed at latest follow-up. None of the patients was able to abduct their shoulder more than 90°. The mean MSTS Score was 63.6% in group P and 63.0% in group B. These differences were not statistically significant (see Table 3). In the present study, 14 patients (8 patients in group P and 6 patients in group B) died of their underlying disease, all other patients were alive without evidence of disease at latest follow-up. Consequently, the disease-specific survival of patients in group P was 74.5% at 5 years and in group B was 67.0% (Figure 8).

## Discussion

The optimal reconstructive technique after proximal humerus resection is controversial. There are several literatures compared the outcome, complications and survival of different type of surgical reconstructions after proximal humeral tumour resection [1-11]. Van de Sande et al.’s [5] study showed that an endoprosthetic reconstruction after proximal humeral resection resulted in the lowest complication rate, highest implant survival and comparable functional results when compared to allograft-prosthesis composite and osteoarticular allograft use. Potter BK and his colleagues [1] stated that implant revision was more common after osteoarticular reconstruction while function was more satisfactory after allograft-prosthetic composite reconstruction. In the study, we have been able to show that prosthetic reconstruction and reconstruction with recycled pasteurized autograft are similar in terms of their local recurrence and metastasis, while the incidence of revisions was higher for patients with prosthetic reconstruction.

In our previous study [14], we treated malignant bone tumors of the distal tibiae with “en bloc” intra-articular excision and ankle arthrodesis using recycled pasteurized autograft, the nonunion rate was 54.5% (6/11). In the present study, the nonunion rate was 25.0% (4/16). Although the characteristics of these two groups are not quite equal to compare, the nonunion rate in the present study was lower, which may be attributed to the recycled pasteurized autograft combined with a non-vascularised fibula autograft. However, the nonunion rate was much higher than other reports. Potter Bk et al. [1] reported only one patient (5.9%) developed nonunion in 17 patients with osteoarticular reconstruction after humeral tumor excision. In van de Sande et al’s study [5], the nonunion rate was 15.4% (2/13). In Aponte-Tinao LA et al.’s reported [4], there were 21 patients with proximal humerus osteoarticular allograft after tumor excision and none became nonuion.

We found 5-year Kaplan-Meier implant survival estimates, with revision for any reason as the end point, of 80.6% and 68.8% for the group P and group B, respectively. In previous reports of osteoarticular graft survivorship, Getty and Peabody [6] found 68% Kaplan-Meier graft survival at 5 years, and Potter et al. [1] reported 56% Kaplan-Meier graft survival at 5 year. However, the 5-year Kaplan-Meier prothesis survival was 100% in Potter et al’s study [1], which was much higher than ours.

In Manfrini et al. study [15], they suggested that free fibular flaps will incorporate into the allograft. Li et al. [16] observed that abundant callus originated from the outlayer of the fibula that union the fibula with the host bone and allograft together. Pasteurized autogenous bones are similar to allografts, in that both possess bone induction ability and bone conductive ability [14]. Sugiura et al. [17] reviewed pasteurized intercalary autogenous bone graft combined with a fibula graft and stated that the addition of a fibula graft seems to promote the theoretically anticipated remodeling process. Our results indicate pasteurized intercalary autogenous bone graft combined with a fibula graft is a useful reconstruction method for large bone defects after resection of osteosarcoma in the humors.

We note several limitations in this study. First, this study was retrospective and the study period included a relatively broad time frame. As such, patient selection between each group was not standardized. Reconstructions with recycled pasteurized autograft generally were performed earlier during the study period as we became increasingly aware of the high nonunion rate associated with this technique. Second, the numbers of patients in each cohort and the study as a whole are relatively small but these numbers are comparable or greater than in previous reports. Third, though only five patients developed local recurrence and eight patients developed pulmonary metastasis at current follow-up, a longer follow up is required since these were malignant lesions with a possibility of late disease recurrence. Fourth, a non-vascularised fibula autograft not a vascularised fibula autograft was adopted in the study, a vascularised fibula autograft maybe better for bone union.

## Conclusions

The study could show that prosthetic reconstruction and reconstruction with recycled pasteurized autograft are similar in terms of their local recurrence and metastasis, while the incidence of revisions was higher for patients with prosthetic reconstruction.

## Competing interests

The authors declare that they have no competing interests.

## Author contribution

TL accountable for the execution of the research, the integrity and analysis of the data, and the writing of the manuscript. QZ accountable for the conception and execution of the research. XG accountable for the integrity and analysis of the data. XZ accountable for the conception of the research. ZL accountable for the writing of the manuscript. XL accountable for the analysis of the data. All authors read and approved the final manuscript.

## Pre-publication history

The pre-publication history for this paper can be accessed here:

http://www.biomedcentral.com/1471-2474/15/69/prepub
